# Ocular Drug Delivery: Role of Degradable Polymeric Nanocarriers for Ophthalmic Application

**DOI:** 10.3390/ijms19092830

**Published:** 2018-09-19

**Authors:** Cheng-Han Tsai, Peng-Yuan Wang, I-Chan Lin, Hu Huang, Guei-Sheung Liu, Ching-Li Tseng

**Affiliations:** 1 Graduate Institute of Biomedical Materials & Tissue Engineering, College of Biomedical Engineering, Taipei Medical University, Taipei 11031, Taiwan; m825105004@tmu.edu.tw; 2 Center for Human Tissues and Organs Degeneration, Institute of Biomedicine and Biotechnology, Shenzhen Institutes of Advanced Technology, Chinese Academy of Sciences, Shenzhen 518055, China; py.wang@siat.ac.cn; 3 Department of Chemistry and Biotechnology, Swinburne University of Technology, Hawthorn, VIC 3122, Australia; 4 Department of Ophthalmology, Shuang Ho Hospital, Taipei Medical University, New Taipei City 23561, Taiwan; ichanlin@gmail.com; 5 Department of Ophthalmology, School of Medicine, College of Medicine, Taipei Medical University, Taipei 11031, Taiwan; 6 Aier Eye Institute; Aier School of Ophthalmology, Central South University, Changsha 410008, China; huanghu@aierchina.com; 7 Menzies Institute for Medical Research, University of Tasmania, Hobart, TAS 7000, Australia; 8 Ophthalmology, Department of Surgery, University of Melbourne, East Melbourne, VIC 3002, Australia; 9 Department of Ophthalmology, Jinan University, Guangzhou 510632, China; 10 Institute of International PhD Program in Biomedical Engineering, College of Biomedical Engineering, Taipei Medical University, Taipei 11031, Taiwan; 11 International PhD Program in Cell Therapy and Regenerative Medicine, College of Medicine, Taipei Medical University, Taipei 11031, Taiwan

**Keywords:** ocular, nanoparticles, polymeric, drug/gene delivery, biodegradable, anterior, posterior

## Abstract

Ocular drug delivery has been a major challenge for clinical pharmacologists and biomaterial scientists due to intricate and unique anatomical and physiological barriers in the eye. The critical requirement varies from anterior and posterior ocular segments from a drug delivery perspective. Recently, many new drugs with special formulations have been introduced for targeted delivery with modified methods and routes of drug administration to improve drug delivery efficacy. Current developments in nanoformulations of drug carrier systems have become a promising attribute to enhance drug retention/permeation and prolong drug release in ocular tissue. Biodegradable polymers have been explored as the base polymers to prepare nanocarriers for encasing existing drugs to enhance the therapeutic effect with better tissue adherence, prolonged drug action, improved bioavailability, decreased toxicity, and targeted delivery in eye. In this review, we summarized recent studies on sustained ocular drug/gene delivery and emphasized on the nanocarriers made by biodegradable polymers such as liposome, poly lactic-co-glycolic acid (PLGA), chitosan, and gelatin. Moreover, we discussed the bio-distribution of these nanocarriers in the ocular tissue and their therapeutic applications in various ocular diseases.

## 1. Introduction

**Figure 1:**
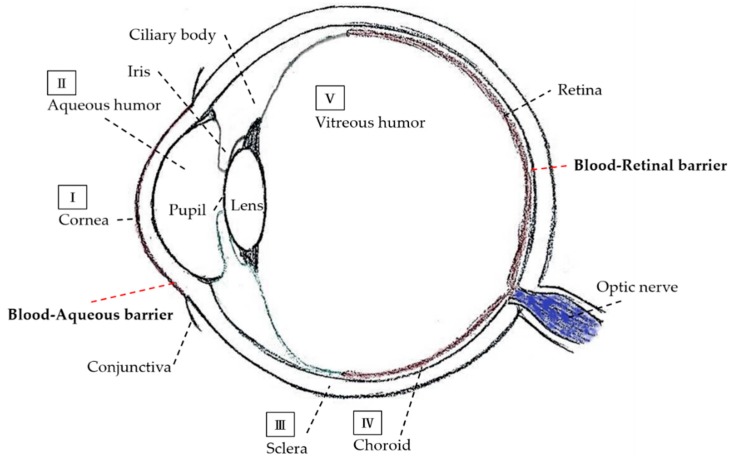
Schematic diagram of the ocular structure with various ocular barriers. The ocular barriers in the anterior segment area (**I**) tear film and corneal epithelium, and (**II**) aqueous humor. The ocular barriers in the posterior segment are (**III**) sclera, (**IV**) choroid, and (**V**) vitreous humor. There are two BRBs. The blood–aqueous barrier in the anterior segment, a part composed of the non-pigmented ciliary epithelial cells and iris capillaries endothelial cells. The BRB, a tight-junction between non-fenestrated capillaries of the retinal blood circulation and retinal pigment epithelial cells in the posterior segment of the eye.

The World Health Organization (WHO) announced that the total population worldwide in 2017 was around 7.5 billion, of which 253 million people suffer from vision impairment and 36 million are blind (4.8%) [1]. More than 80% of people are aged 50 years or older [1]. Vision loss and blindness are major health problems that cannot be ignored in the elderly population. The eye is the organ of the visual system and an important tissue for vision. It is a globular structure with a diameter of 24 mm, and a mass of approximately 7.5 g in humans. From a lateral view of the eyeball (see Figure 1), the cornea is located at the outer anterior segment of the human eye, followed by the anterior chamber, pupil, iris, lens, and conjunctiva [2]. The posterior segment of the human eye includes the vitreous humor, retina, macula, optic nerve, choroid, and sclera [2]. The retina plays a vital role in fine detailed visual acuity and color vision. The primary function of the retina is to process visual information as well as control image formation. The retina is a thin and light-sensitive tissue of approximately 0.5 mm thickness with multiple cell layers including the ganglion layer, inner plexiform layer, inner nuclear layer, outer plexiform layer, outer nuclear layer, photoreceptor layer, and retinal pigment epithelium from the direction of light entry [3]. The choroid is a vast network of capillaries which supply nutrients to the retina in the human eye through the central retinal artery and the choroidal vessels with the greatest blood flow (65–85%). The eye is a slow blood circulation organ with many physiological barriers (Figure 1), meant to keep the systemic circulation separate from ocular tissues. Furthermore, the central nervous system, including the eye, brain, and spinal cord, is believed to be sealed from the circulation [4], and thus the eye is considered ‘immune privileged’. The anatomical and physiological barriers of the eye make it a highly protected organ shielded from the systemic circulation. Therefore, when an ocular disease occurs, it is difficult to treat with medications, especially in the posterior segment of the eye [5,6]. Currently, several drug delivery modalities such as intravitreal injection, which is the gold standard method for posterior drug delivery, have been applied for treating posterior ocular disease. Subretinal injection, subconjunctival injection, and topical administration are also used. However, these are not satisfactory, thus a better approach still needs to be further explored [7].

The current treatment modality for most ocular diseases requires frequent intraocular injections, with the concomitant risks associated with any invasive intraocular procedure. A non-invasive drug delivery route could potentially eliminate the risks of injection into the eyes. However, non-invasive drug delivery routes, such as topical delivery, have been a significant challenge due to the unique anatomy and physiology of the eye. The invasive treatments include surgery, laser therapy, frozen therapy, and drug administration by intraocular or periocular injection. Surgery, laser, and frozen therapy can prevent disease deterioration, but with high recurrence rates [8]. The intraocular or periocular injection delivery methods include subconjunctival, intravitreal, and subretinal injections. These often require frequent injections to achieve therapeutic effects in the eyes and are usually accompanied by complications, such as inflammation, high intraocular pressure, cataract, retinal hemorrhage, and even retinal detachment [9,10]. Although intravitreal injection is currently a standard method for posterior ocular drug delivery, the complications mentioned above may carry risks of potential visual loss. Therefore, each treatment has its drawbacks or challenges that must be overcome, and there is also an urgent need to develop a new therapy for increasing posterior ocular diseases treatment such as glaucoma, diabetic retinopathy, and age-related macular degeneration (AMD) [11].

Drug administration through non-invasive pathways, including oral medications, eye ointments and topical eye drops, have been widely used to treat various eye diseases, but most of them are ineffective, and only applicable to early mild symptoms [12]. Moreover, the physiological barriers of the eye often limit the bioavailability of these non-invasive treatments. For instance, the blood–retinal barrier (BRB) impedes the oral administration from getting into the systemic circulation [13], and the corneal epithelial barrier reduces the drug concentration in the eye when ointments and eye drops are administered on the ocular surface. The topical eye drop is rapidly removed from the ocular surface leading a short drug retention time. Typically, less than 5% of the drug administered is retained on the ocular surface as a result of the corneal epithelium barrier and nasolacrimal duct drainage [13,14]. Although these treatments were more acceptable to patients, the poor bioavailability due to ocular barriers results in difficulties for topical drug delivery to the cornea and retina [15].

### 1.1. Barriers in the Anterior Part of the Eye

After topical instillation of a drug, the first and outermost barrier of the eye is the tear film on the ocular surface. The flow of lacrimal fluid moves the drug to the nasolacrimal duct from the ocular surface in a few minutes. The lacrimal turnover rate is approximately 1 μL/min. This tear drainage mechanism results in the poor drug bioavailability of topical delivery [13,14,16]. Another barrier is the cornea, as shown in Figure 1I, which is approximately 500 μm thick. The healthy cornea is a transparent, clear, and avascular tissue consisting of five layers including the corneal epithelium, Bowman’s layer, corneal stroma, Descemet’s membrane, and corneal endothelium [6,17]. The corneal epithelium is lipophilic in nature with tight junctions, which leads to limitation of the permeation of hydrophilic molecules. The highly organized corneal stroma consists of collagen fibers, closely ranged together. It is not only an effective barrier to most microorganisms but also for drug absorption. The innermost layer of the cornea is the corneal endothelium, which is a monolayer of hexagonal endothelial cells to adjust water influx into the cornea and a barrier between the cornea and aqueous humor (Figure 1II). These characteristics make the cornea a major barrier, and a challenge for drug delivery to the anterior segment of the eye [17,18]. 

The conjunctiva is a mucous membrane consisting of vascularized epithelium, located at the posterior surface of the eyelids and outer area of the cornea, which is involved in the formation and maintenance of the tear film, and also protects the ocular surface from environmental pathogens [19]. Both corneal and conjunctival epithelia have tight junctions that restrict the entrance of substances into the eye. Besides, the mucus layer in the eye blocks the entrance of not only particles but also medicines, which are then removed through the lacrimal system. The other obstruction to drug delivery in the anterior part of the eye is the blood–aqueous barrier (BAB), shown in Figure 1. The BAB includes the ciliary epithelium and capillaries of the iris [3] and is composed of non-pigmented ciliary epithelial cells of the ciliary body and endothelial cells in the iris’s vessels. The function of the BAB is preventing unfettered passage of molecules from iridial vessels [20].

### 1.2. Barriers in the Posterior Part of the Eye

The sclera, which surrounds the outermost layer of the eye’s globe (Figure 1III), connects the anterior and posterior parts of the eye. It is composed of extracellular matrix including collagen fibrils and glycoproteins to maintain the ball shape. The sclera is easily permeable to hydrophilic molecules. The choroid is a pigmented middle layer between the sclera and retina, as shown in Figure 1IV, and is a highly vascularized coat covering 80% of the posterior external segment of the eye. The choroid also contributes to maintaining the ocular equilibrium and intraocular pressure (IOP), since it provides the blood containing oxygen and nutrition to the outer retina as well as the retinal pigmented epithelial (RPE) layer [21]. 

As shown in Figure 1V, the vitreous body (about 4 mL volume) is composed mainly of a gel structure in water (99%); non-collagenous proteins (fibrillin-1, opticin, and VIT1); types I, V, IX, XI collagens; hyaluronic acid (HA); proteoglycans of chondroitin sulfate; and heparan sulfate [22]. The major function of the vitreous body is to maintain ocular completeness and transport nutrients between the retina [22]. Since the vitreous humor is filled with viscous gel, the diffusion of molecules from the vitreous humor to the retina is limited greatly. The big and charged molecules are difficult to transport to retina, due to their aggregation behavior and may interact with negatively charged HA and anionic collagens and finally cause molecules to precipitate in the vitreous humor [23].

The blood–retinal barrier (BRB), shown in Figure 1, is a specialized transport barrier between the blood and the retina and has tight junctions between the monolayer of RPE cells (outer part of BRB) and retinal capillary endothelial cells (inner part of BRB) of the retinal circulation [24]. As a result of the anatomic position of the BRB, it effectively limits the transportation of molecules from the choroidal blood circulation to the posterior segment of the eye [25]. Moreover, the BRB also plays an important role in controlling the environment of the neural retina compared to the high blood flow and leaky walls of choroidal vasculature where molecules easily enter into the choroidal extracellular gap, but have difficulty passing through the RPE layer which is a firmly tight monolayer limiting the transportation of molecules [20].

## 2. Methods for Ocular Drug Delivery

The physical barriers and blood–ocular barriers mentioned above are primary obstacles to limiting ocular drug delivery, and how to overcome these barriers is a major challenge in ophthalmic drug development. Barriers in ocular anatomy and physiology are inherent and unique, which can protect the eye from the invasions of environmental toxicants and microorganisms. The blood–ocular barrier also separates the interior portion of the eye from the blood circulation into the eye; however, it also limits the bioavailability of drug during systemic administration [25]. 

**Figure 2:**
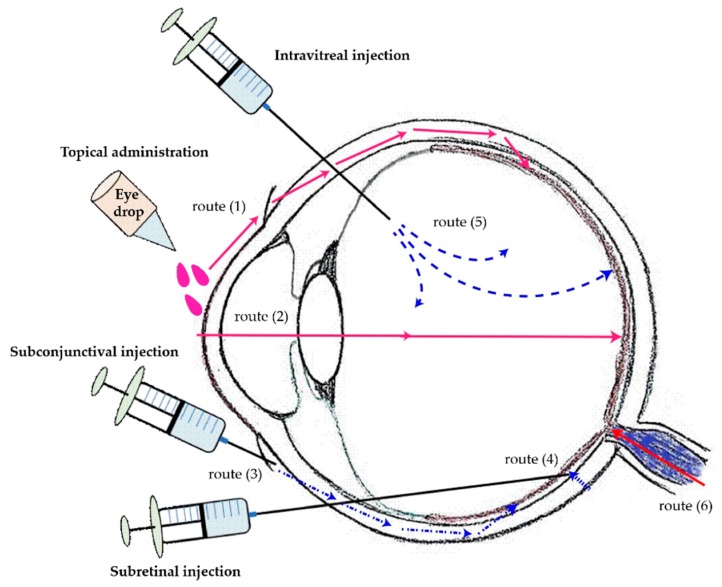
Methods of ocular drug administration and its delivery routes to the posterior segment. Routs of drug transportation to the back of the eye via topical administration (1 and 2), subconjunctival injection (3), subretinal injection (4), and intravitreal injection (5). The drug transportation from the systemic circulation via oral medication (6).

For anterior drug delivery, eye drops, or ointment formulations are often used, but not for the posterior part of the eye. As shown in Figure 2, there are some common approaches to deliver ophthalmic medications to the posterior area of the eye. The major obstruction of retinal drug delivery for systemic and topical eye drop administration are the physiological barriers such as the BRB and corneal epithelium in the eye [26]. There are two pathways to deliver drugs to the posterior ocular segment by topical administration (eye drops): firstly (Route 1), the drug diffuses to the conjunctiva from the ocular surface, then penetrates the sclera pore to the choroidal circulation and the posterior choroid, and finally reaches the RPE layer from the choroidal vessels. Second (Route 2), the drug penetrates the eye through the corneal surface, anterior aqueous chamber, lens, and reaches the vitreous body; then, the drug diffuses to the inner limiting membrane, then reaches inside the retina. The subconjunctival injection delivery route (Route 3). After injection, drugs penetrate through the sclera pores to the choroidal circulation and the posterior choroid lately; and then get to the RPE layer from the choroidal vessels. Route 4 represents subretinal injection. The drug is injected into the posterior ocular segment directly and subsequently diffuses to the RPE layer and the inner retina. The most used way for posterior ocular drug delivery is intravitreal injection, shown as Route 5. The drug is injected into the vitreous humor, then diffuses in various directions, and crosses the inner limiting membrane into the retina. Due to the complexity of the three-dimensional network of collagen fibrils bridged by proteoglycan filaments in the vitreous body, the efficacy of retinal drug delivery by intravitreal injection is significantly impaired [22]. Also, the colloidal state of the vitreous humor prevents the drug from penetrating into the retina and results in a poor bioavailability of the drug. Even if the drug can reach the retina, there is an internal limiting layer as a barrier to prevent drug penetration into the retinal cells [27,28]. Lastly is Route 6, the drug reaches the RPE layer from the systemic circulation via oral medication. Oral medications have a certain chance of delivering the drug into the posterior segment of the eye; however, it is difficult to achieve an effective dose in some cases.

## 3. Advantages of Nanocarriers for Ocular Drug Delivery

Recent advances in nanotechnology provide novel opportunities to overcome the limitations of conventional drug delivery systems through the fabrication of nanostructures capable of encapsulating and delivering small molecules. Nanoparticles are described as materials with a length of 1–1000 nm in at least one dimension; By strict definition, nanomaterials are objects in the range of 1 and 100 nm and exhibit dimension-dependent phenomena such as the quantum-size effect [29]. However, by generalized definition, nanoparticles with drug loading have small sizes ranging from 1 to 1000 nm and can be fabricated through chemical processes to control the release of therapeutic agents and enhance their penetration through different biological barriers of the eye [29,30]. According to previous studies of ophthalmological applications, the size of complex drug particles should be less than 10 μm to avoid a foreign body sensation after administration [31]. Especially for ocular drug delivery, larger sized particles (>1 μm) may potentially cause ocular irritation [32]. Based on these results, delivery of ocular therapeutics via nanoparticles can be used to reduce the sensation and irritation of the eye. The main advantages of using nanocarriers in the treatment of ocular diseases are to enhance bioavailability of topical administration, achieve controlled release, targeted delivery, and ultimately improved therapeutic efficacy [25,33]. Moreover, studies have shown that drug-loaded nanocarriers (nanomedicine) for treating anterior ocular diseases have the advantages of lower dosage requirements, high drug retention rate, less dosing frequency, and high patient tolerance and acceptance. These factors reveal the potential of nanomedicine to replace traditional eye drops as a primary option for anterior ocular therapy in the near future [34,35].

### 3.1. Nanocarriers Can Overcome the Ocular Barriers

In recent years, several types of nanocarriers have been explored for ocular drug delivery especially degradable nanoparticles (NPs) made with polymers, such as liposome, dendrimer, chitosan nanoparticle, poly lactic-co-glycolic acid (PLGA) nanoparticle, and gelatin nanoparticles. These studies suggest that properties of nanocarriers could influence their ophthalmic application in the anterior or posterior segment of the eye [36].

#### 3.1.1. Surface Charge of Nanoparticles Influence Ocular Tissue Interaction

In the anterior segment of the eye, scientists have made significant contributions to improving the efficacy of treatments for ocular diseases by enhancing the duration of drug retention on the ocular surface and increasing drug bioavailability [17,36]. For instance, the cornea and conjunctiva possess negative surface charges, and it is expected that the cationic colloidal NPs can enhance the retention time on negatively charged ocular tissues more efficiently than the anionic carriers, providing an increased opportunity for the drug to enter the eye [37]. Tseng et al. 2013, proved that the topical administration of positively charged gelatin nanoparticles could prolong the drug retention time on the negatively charged ocular surface, compared to the free-form drug formulation [38]. Xu et al. 2013, found that NPs coated with different surface charges of polyethylene glycol (PEG) resulted in a variant delivery efficacy in an ex vivo model of the bovine vitreous body. Since negatively charged HA and glycosaminoglycan proteins exist in the vitreous body, those particles with positive charges were fixed in the vitreous humor due to electrical attraction; however, the negatively charged particles can diffuse through the vitreous body to deeper sites of the eye [39]. Similarly, Ying et al. 2013, demonstrated that the surface charge has a great influence on intraocular drug transportation when submicron-sized lipid emulsion is delivered to the retina [40]. This evidence suggests that the surface charge of the NPs is a key factor in determining their distribution in different regions of the eye [36,41]. Besides, Koo et al. reported that the modified amphiphilic NPs could overcome the physical barrier of the inner limiting membrane and improve the penetration into the deeper retina after intravitreal injection [42]. Their study also indicates that intravitreal NP activity relies on the charged surface to permit the vitreous diffusion and the penetration into the deeper retina. Another study reported by Kim et al. 2009 found that cationic NPs of human serum albumin (HSA) interacted with the negatively charged glycosaminoglycans in the vitreous, consequently impeding their diffusion in the vitreous and penetration into the retina. Conversely, anionic HSA NPs tend to diffuse in the vitreous before they penetrate into the retina. In this study, authors also emphasize that the vitreous acts as a static barrier that limits drug delivery to the posterior segment and illustrates the role of NP surface charge in hindering or facilitating the diffusion across the vitreous and into the retina [43].

#### 3.1.2. Size Effect of Nanoparticles for Penetrating into Ocular Tissue

The size of NPs is also a key factor in ocular drug delivery. In order to achieve an effective drug delivery, NPs need to be small enough in size to penetrate the ocular barriers [31]. Hagigit et al. 2012 showed that cationic nano-emulsion containing 1,2-dioleoyl-3-trimethylammonium-propane chloride (DOTAP), of size around 95 nm and zeta potential about +56 mV, can effectively permeate the cornea and the conjunctiva of a male albino rat eye through topical instillation [44]. Moreover, eye drop formulations containing gelatin nanoparticles (GPs), around 180 nm showed a wide distribution in rabbit corneal cryosection and can be retained for a longer time by uptake into cornea epithelium cells [38]. The frequency of drug administration can also be reduced by the long-term release effect of NPs in the treatment of retinopathy and posterior ocular diseases. Indeed, various synthetic NPs (chitosan, liposomes, PLGA, HA, albumin, etc.) have been explored for drug delivery to the retina via intraocular injection [45,46,47]. In general, NPs less than 250 nm are usually taken up by endocytosis [48,49]. Nanoparticles in the range of 50–350 nm possessing positive charges can be transported or diffused through the vitreous body after intravitreal injection. When NPs are <350 nm, the charge effect is the major factor influencing its distribution; lager then that, the size effect may play a major role. Jo et al. 2011, reported that NPs could cross through BRB or other ocular barriers. [50]. A recent study reported by Bisht et al. 2018 also showed that the size of scleral water channels/pores is about 30 to 300 nm. Nanoparticles smaller than these pores will be able to pass through the scleral barrier and then diffuse into the vitreous humor [51]. 

**Figure 3:**
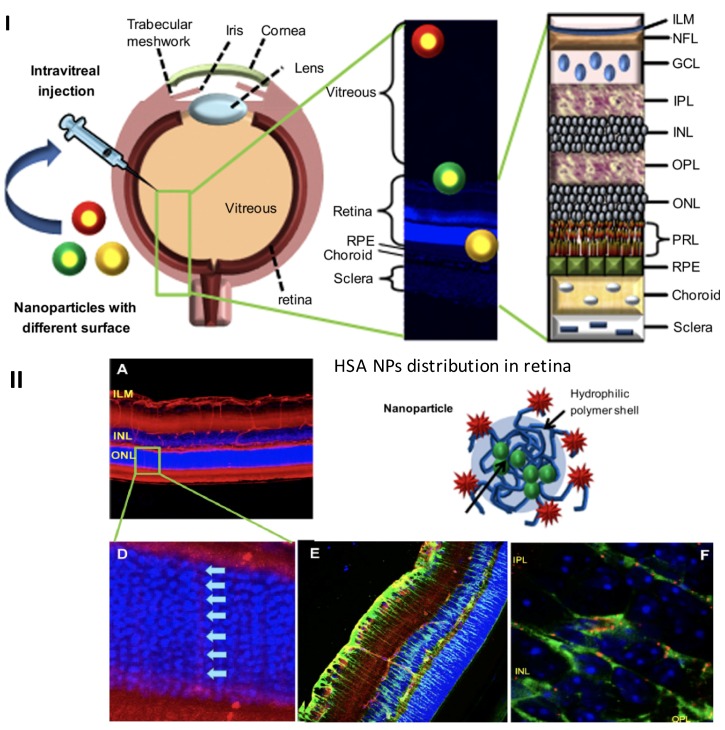
(**I**) Distribution of NPs with various surface properties in the different region of vitreous and retina via intravitreal injection. (**II**) Distribution of HSA NPs (red channel) in the retina via intravitreal injection. (**A**,**D**) Penetration of HSS NPs into the retina at 6 h post-injection, (**E**) scan of the whole retina, and (**F**) colocalization with Müller cells (green channel). ILM: inner limiting membrane, NFL: nerve fiber layer, GCL: ganglion cell layer, IPL: inner plexiform layer, INL: inner nuclear layer, OPL: outer plexiform layer, ONL: outer nuclear layer, PRL: photoreceptor layer, RPE: retinal pigment epithelium. Image adapted from Koo et al. (2012) and reprinted with permission from Biomaterials (Koo et al. 2012) [42].

How the size and charge of NPs influence their interaction with ocular tissues is summarized in Table 1. A previous study has shown that size and surface charge affect molecular permeation through the sclera layer simultaneously; the large and positively charged molecules experience more difficulty entering the sclera and may also be captured by the negatively charged glycoproteins [23]. Several studies have further explored the impact of size and surface charge of NPs on ocular penetration after intravitreal injection. Variant NPs, the size of 230–350 nm but differing in surface properties, were tracked with fluorescent dyes for their delivery from the vitreous to the retina after intravitreal injection (Figure 3I) [42,43]. Polyethyleneimine (PEI) NPs with strong positive charges (+33.5 mV, 316 nm) were found to aggregate spontaneously before reaching the retina. Hyaluronic acid-based NPs do not form aggregations in the vitreous due to their firm negatively charged surfaces (−26.2 mV, 213 nm), and most of these NPs penetrate the retina and enter the RPE cell layer (Figure 3I) [42]. Hybrid combinations of NPs exhibit surface properties reflecting their constituents. For instance, HSA/glycol chitosan hybrid NPs (−1.9 mV, 293 nm) was found to accumulate in the internal limiting membrane and were unable to penetrate into deeper retinal structures. Interestingly, due to the pore size of the external limiting membrane (3 and 3.6 nm), another type of NP, HSA NPs (−20.6 mV, 326 nm), is not expected to overcome this barrier. However, it was found that HSA NPs penetrated all retinal layers and quickly reached the outer retinal structures, including the photoreceptor and RPE layers [43]. Although HSA NPs are larger in molecular size, strong negative charges enhance their specific targeting and penetration of both the nuclear layer and the outer plexiform layer (Figure 3II) [42].

## 4. Polymeric Colloidal Nanocarriers for Ocular Drug Delivery

Due to the chronic nature of many ocular diseases and the unique anatomical location with a barrier-filled environment in the eye, drug treatment usually requires frequent dosing. Biodegradable polymeric NPs can serve as suitable nanocarriers for solving the problem of frequent administration and protect the drug from contact with enzymes/proteins in the circulation, thereby increasing its half-life. Drugs carried by NPs can also be sustained with controlled release at the desired area to reduce the need for frequent dosing. By modifying size/charge, the delivery properties of the NPs can be manipulated to target the desired region in the anterior/posterior region of the eye. The small size of these NPs can also help to overcome the blood–ocular barriers [25,52]. In the following section, we summarize the application of various biodegradable polymeric nanocarriers in ocular drug/gene delivery. 

### 4.1. Liposome (Lipid)

Liposomes are tiny round shape bubbles with a phospholipid bilayer structure like a cell membrane, and is suitable to carry hydrophilic or lipophilic drugs. Liposome NPs are the most popular and well-studied vehicles for drug delivery. Karn et al. 2014, developed the cyclosporine A (CsA)-encapsulated liposomes for dry eye syndrome (DES) treatment [53]. In this study, the male albino rabbits were induced into DES and then treated with CsA-liposomes by topical delivery (eye drops) compared with a commercially available CsA emulsion (Restasis^®^). The results indicate that the CsA-liposomes result in lower ocular irritation and better therapeutic efficacy with higher tear amount compared to the non-liposomes group. De Sá et al. 2015, used liposomal voriconazole (VOR) for fungal keratitis treatment via topical administration [54]. VOR can be effectively encapsulated in liposomes and can penetrate into bovine or porcine cornea ex vivo, releasing a fair amount of 47.85 ± 5.72 g/cm^2^ VOR into the cornea 30 min after instillation. Besides anterior ocular drug delivery, liposome-based eye drops formulations have also been applied in posterior ocular delivery. Davis et al. 2014, reported a topical application of the Annexin A5-associated liposomes with Avastin encapsulation for drug delivery to the posterior ocular segment [55]. The study showed that the Annexin A5-liposomes overcome biological barriers such as the corneal epithelial barriers in rats and rabbits, and then successfully deliver Avastin to the posterior segment of the eye with the final concentration of 127 ng/g acquired from rat eyes and 18 ng/g from rabbit retina after topical administration. Lajunen et al. 2014, developed the plasmid DNA encapsulated liposomes by using microfluidizer production [56]. With transferrin (Trf) modified on the liposome surface, it exhibits high penetration and targets the RPE by topical instillation. The author also examined the size-dependent effect of these Trf-modified liposomes. Compared with non-Trf modified liposomes, diameters less than 80 nm (68 nm and −36 mV) penetrated the RPE layer, and 100 nm (100 nm and −36 mV) or larger were distributed in the choroidal endothelium. These results indicate the size-dependent effect of liposomes distributed in different areas of the posterior segment of the eye. This study also demonstrated that ligand-modified liposomes have the potential to be used as drug carriers for the treatment of retinal diseases by topical instillation.

Natarajan et al. 2012, developed latanoprost-loaded egg-phosphatidylcholine (EggPC) liposomes (the size of 109 nm and drug loading efficacy of 94%) for reducing IOP [57]. It was delivered to the subconjunctival space in the superior temporal region of rabbit eye by a single subconjunctival injection, latanoprost was sustainably released in the rabbit eye for up to 90 days, and no adverse side effects were found. Intraocular pressure reduction was observed with a daily topical instillation of latanoprost (reduction: 2.5 ± 0.9 mmHg); however, the single subconjunctival injection of latanoprost-loaded EggPC liposomes showed a greater effect in lowering IOP (reduction: 4.8 ± 1.5 mmHg) at 90 days in the rabbit eye. Clearly, subconjunctival delivery of liposomes can bypass ocular barriers, thereby allowing these nanocarriers to be potentially used as a delivery platform for the sustained release of drugs in the treatment of glaucoma [57]. Besides, Zhang et al. 2010, evaluated the therapeutic effect of tacrolimus (FK506) encapsulated in liposomes in experimental autoimmune uveoretinitis (EAU) in rats via intravitreal injection [58]. After intravitreal injection, tacrolimus (FK506)-encapsulated liposomes were located in the vitreous body and internal limiting membrane of the retina. Liposomes had migrated from the internal limiting membrane to the outer nuclear layer at 24 h, and reached the retina 7- and 14-days post-injection. Moreover, their results also showed that tacrolimus could still be detected in the ocular fluids 14 days after injection (the concentration was higher than 50 ng/mL) and significantly reduce intraocular inflammation without causing any side effects on retinal function as well as immune rejection. Bevacizumab (Avastin^TM^) is a large molecular weight (149 kDa) recombinant humanized monoclonal antibody that blocks neovascularization by neutralizing human vascular endothelial growth factor (VEGF). Abrishami and colleagues synthesized the bevacizumab-loaded liposomes for intravitreal delivery. The bevacizumab-loaded particles were prepared using phospholipid and cholesterol to form multilamellar liposomes in a 1:1 molar ratio. The efficiency of bevacizumab encapsulated was 45% and it remained stable after liposomal process. Although authors claim the bevacizumab loaded liposome is nanosized, data of particle size was not shown. Intravitreal injection of bevacizumab-loaded liposomes in the rabbit eyes showed a higher drug concentration-time curve and a slower clearance compared to the antibody solution [59].

### 4.2. Chitosan (Polysaccharide Based) Nanoparticles

Chitosan is a polysaccharide copolymer comprised of glucosamine and N-acetylglucosamine. It can be obtained by deacetylation of chitin from crustacean shells, with different molecular weights (50–2000 kDa), viscosities and degrees of deacetylation (40–98%) [60]. The advantages of using chitosan drug carriers are their low production cost, biodegradability, biocompatibility, and U.S. Food and Drug Administration (FDA) approved biomaterial. Especially as the drug carrier for ocular drug delivery, chitosan has excellent tolerance and penetration of the corneal surface due to its mucoadhesive property and ability to open tight junctions [61].

Many studies have explored the application of chitosan NPs for ophthalmic drug delivery. For example, Nagarwal et al. 2010, developed the 5-fluorouracil (5-FU)-loaded chitosan NPs (CH-DNPs) for ocular delivery [62]. The size of CH-DNPs is in the range of 192 nm with a zeta potential of 42 mV positivity. After instilling CH-DNPs into the cul-de-sac of rabbit eye eyelids, no sign of irritation and inflammation were observed on the ocular surface. The in vivo 5-FU concentration in the aqueous humor of CH-DNPs-treated eyes was higher compared to the free-form 5-FU solution-treated eyes. The prolonged retention time of 5-FU in the precorneal area resulted from the mucoadhesive characteristic of chitosan within the tear film. Chitosan NPs are also used to deliver anti-infective agents for the treatment of bacterial infections in the eye to overcome the difficulties of penetrating the innate protective barriers in the ocular surface. Silva et al. 2015, prepared chitosan NPs with daptomycin (a natural lipopeptide antibiotic) encapsulation against Gram-positive bacteria for the treatment of intraocular infections such as endophthalmitis [63]. In this study, the antimicrobial activity of daptomycin was preserved when the antibiotic was encapsulated into chitosan NPs. Authors further indicated that chitosan NPs have a high antibacterial ability because the polycationic structure of chitosan can bind to negatively charged bacterial cells through a higher positive charge, destroying the bacterial cell membrane and causing bacterial cell death [63]. 

Chitosan can also be used as a non-viral gene carrier as it has several advantages, including high transfection efficacy, less immunogenicity, and lack of mutational potential compared to virus vectors [62]. Klausner et al. 2010, synthesized the oligomeric chitosan-DNA NPs the size of 98.2 nm with a strong positive charge at 44.1 mV [64]. The efficacy of in vivo transfection was evaluated through the injection of oligomeric chitosan-DNA NPs into rat corneal stroma. After 24 h of transfection, the gene expression in the oligomeric chitosan-DNA NPs-injected group was 5.4-fold greater than that in the group that had received polyethyleneimine-DNA NPs and was only observed in corneal stroma and corneal fibroblasts. Therefore, the authors concluded that this oligomeric chitosan-DNA NPs can be used as a promising drug carrier for the treatment of corneal diseases. In addition, Mitra et al. 2014, developed the glycol chitosan NPs encapsulated plasmid DNA for posterior ocular gene delivery [65]. The results showed that the encapsulation of plasmid DNA into glycol chitosan NPs (size in the range of 330 to 410 nm and surface charged at +24.17 mV.) did not affect its gene expression capacity. After subretinal injection in adult mice for 14 days, a significant amount of green fluorescent protein (GFP) proteins were expressed in the RPE layer of the eyes that had received glycol chitosan NPs-loaded GFP plasmid DNA compared to the eyes that had received naked plasmid DNA or saline. Electroretinogram further noticed no effect on the retinal function after 30 days of injection. These results suggest that glycol chitosan NPs have excellent biocompatibility and high transfection efficacy, which are well suited to be gene carriers for the treatment of RPE-associated genetic diseases. Moreover, the biodegradability of chitosan in living organisms depends on the molecular weight and degree of deacetylation. The hydrolyze linkages between glucosamine–*N*-acetyl-glucosamine, glucosamine–glucosamine, and *N*-acetyl-glucosamine–*N*-acetyl-glucosamine of chitosan can be degraded by enzymes such as lysozyme. And, degradation of chitosan can be carried out by chitosanase, chitin deacetylase, and β-*N*-acetylhexosaminidase [66,67]. Slow release of the drug due to degradation is one of the benefits of using chitosan-based nanocarriers.

### 4.3. PLGA Nanoparticles

Poly lactic-co-glycolic acid (PLGA), a copolymer of poly lactic acid (PLA) and poly glycolic acid (PGA), has been successfully developed and used in medical applications such as surgical sutures, bone plate/screws, tissue engineering scaffold, and drug carrier systems [68,69], which is an FDA approved biodegradable material. Poly lactic-co-glycolic acid is a highly biocompatible, biodegradable, controllable material, with mechanical properties that can be modified by changing the PLA/PGA ratio and molecular weight. PLGA undergoes hydrolysis in vivo to produce biodegradable metabolite monomers, such as lactic acid and glycolic acid, which have very minor systemic toxicity associated with the use of PLGA for drug delivery [70]. PLGA also has superior hydrophilicity and strong physical strength, which make it an excellent controllable drug carrier for medical applications [71]. Several advantages have been noticed by the use of PLGA based-NPs for ophthalmic drug delivery, including protection of encapsulated drugs from rapid inactivation, maintenance of slow drug release due to polymer degradation, and targeting of specific regions or cells by surface modification. Moreover, PLGA NPs have a high encapsulation efficiency for hydrophilic or hydrophobic drugs, even macromolecules, proteins, peptides, and nucleic acids [72].

Cañadas et al. 2016, evaluated the delivery efficacy of PLGA NPs with pranoprofen (PF) (PF-F NPs), a kind of non-steroidal anti-inflammatory drug, encapsulation (PF-F NPs) in the cornea via topical instillation [73]. The size of PF-F NPs is around 350 nm and its surface charge is −7.41 mV with 80% PF encapsulation rate. An in vitro study was performed in the Y-79 human retinoblastoma cell line to evaluate the cytotoxicity of PF-F NPs, and the results showed that blank PLGA NPs were not toxic to the cells and could lower the cytotoxicity of PF. The study also examined the effect of PF-F NPs on ex vivo corneal permeation, in vivo ocular tolerance and anti-inflammatory activity compared to commercial eye drop formulations and free-form drug solutions in rabbits. It was found that the corneal permeation coefficient of PF-F NPs was four times higher than that of other groups. The PF-F NPs with PF loading had a rapid onset of anti-inflammatory action and showed prolonged retention time on the cornea surface, which significantly reduced ocular edema. These results suggest that PF-F NPs are a potential therapeutic alternative for the management of corneal diseases associated with chronic inflammation. Similarly, Salama et al. 2016, also evaluated the therapeutic efficacy of PLGA NPs encapsulated with the anti-inflammatory corticosteroid fluocinolone acetonide (FA-PLGA) via topical installation for treating intermediate uveitis, posterior uveitis, and panuveitis [74]. The size of FA-PLGA ranges from 85 to 160 nm with a negative charge of 5 mV, and the encapsulation efficiency of FA can almost reach 100%. Moreover, to enhance the mucoadhesion ability of FA-PLGA, the surface of the NPs was modified with 0.1% *w*/*v* chitosan. With chitosan surface modification, its size increased to 779.5–1302.5 nm, and zeta potential moved to 1.9 mV. The chitosan-coated PLGA NPs showed a greatly prolonged residence time of NPs on the ocular surface due to the positive zeta potential of chitosan coating. The pharmacokinetic analysis of tears showed that the drug levels were highest 30 min after instillation therefore, chitosan coated PLGA NPs can be ideal nanocarriers for rapid and sustained drug delivery to the cornea [75].

Bisht et al. 2018, encapsulated Connexin43 mimetic peptide (Cx43MP), which is a peptide that inhibits the pathological opening of gap junction hemichannels, into PLGA NPs (Cx43MP-PLGA NPs) and evaluated the capacity of posterior ocular delivery via intravitreal injection [76]. The size of Cx43MP-PLGA NPs is in the range of 75.6–153.8 nm with a zeta potential in the range of (−9.4)–(−46) mV. The study showed that Cx43MP-PLGA NPs is biocompatible since no apoptosis or cellular death was noticed in the zebrafish and even the live embryos. Tahara et al. 2017, investigated the posterior ocular delivery of PLGA modified NPs via topical administration [77]. For enhancing the mucoadhesive property, surface modified PLGA NPs with chitosan, glycol chitosan, or polysorbate 80 (P80) were evaluated. The size of unmodified PLGA NPs is approximately 224.5 nm with a −41.3 mV surface charge. The size of P80-PLGA NPs is similar to the unmodified PLGA NPs. The size of chitosan- and glycol chitosan-PLGA NPs are larger than other groups because the chitosan or glycol chitosan molecules are adsorbed on the PLGA surface. Since chitosan and glycol chitosan are cationic polymers, both chitosan- and glycol chitosan-PLGA NPs were modified to a weak negative charge (−9.34 mV) and a strongly positive charge (39.9 mV) NPs, respectively. After topical administration, it was found that surface modified PLGA NPs with chitosan or glycol chitosan or P80 could penetrate mouse retina [77]. Mucoadhesive molecular modifications can enhance the interactions between PLGA NPs and the cell surface and then improve the retinal penetration. Besides, the in vivo study has also shown that the posterior ocular transport of PLGA NPs may occur through the non-corneal route (from the conjunctiva, periocular tenon tissue, posterior sclera, posterior choroid, to the retina), thereby surface-modified PLGA NPs are a promising carrier for retinal drug delivery via topical instillation [77]. 

### 4.4. Gelatin Nanoparticles

Gelatin is a natural biopolymer prepared and purified from collagen (usually from porcine skin, cow bone, or fish scale) via acid or alkaline hydrolysis. It has a triple helix structure and polyampholyte (both cationic and anionic) property. Gelatin NPs (GPs) have been previously used as drug and gene carriers with reported successful drug/gene delivery in ophthalmic application. Gelatin NPs exhibit an excellent biocompatibility, biodegradability, low cost, and are easy drugs to manufacture [78,79]. The degradability of gelatin is due to the terminal amino residues of gelatin created during collagen hydrolysis, these N-linked amino peptides can be cleaved into amino acid residues. Peptide bonds involving amino groups of serine and threonine are particularly susceptible to acid and base hydrolysis. The aspartic peptide in gelatin is only sensitive to acid hydrolysis. When the enzyme leaves in vitro or in vivo with gelatin, degradation of gelatin is caused by enzymatic digestion. Gelatin peptide segments can form configuration with enzymes, resulting in sensitivity to a range of proteolytic enzymes such as papain, pepsin, chymotrypsin, and trypsin [80]. More importantly, it is an FDA-approved biomaterial. Since collagen is the major component of corneal stroma, the use of gelatin NPs as the drug carrier in eye drop formulations can improve the bioavailability of drugs or genes by interacting with corneal and conjunctival glycoproteins [26,81]. 

Tseng et al. 2013, developed two different charged GPs and evaluated the biocompatibility in the human corneal epithelium (HCE) cells in vitro as well as in the rabbit eye in vivo administrated topically [38]. The positively charged GPs (GP(+)) were prepared with type A gelatin of 180.6 nm and positive charge of 33.4 mV, and the negatively charged GPs (GP(−)) were prepared with type B gelatin of 230.7 nm and surface charge at −44.2 mV. The intracellular GPs(+)/(−) accumulation in HCE cells was confirmed, revealing that the intracellular fluorescence intensity of the cell lysates in the GP(+) group was higher than the GP(−) group after 10 to 60 min cultivation. This result indicated that cationic NPs could increase interaction with cells, consequently increasing bioavailability and transfection efficiency. The GP(+) used as eye drop on rabbit eyes (100 µg/mL, 50 µL) was safe and caused no irritation to the eyes of the all tested rabbits. No influence on corneal thickness and changes in IOP were found as well [38]. In addition, the fluorescence of GP(+) in the cornea was widely distributed and longer drug retention on the corneal surface was observed, possibly due to the positive charge of GP(+) absorbed on the negatively charged cornea. Overall, GPs(+) have a great potential as the drug/gene carriers for the treatment of corneal disease. In another study, Mahor et al. 2016, developed the moxifloxacin-loaded GP (MGP) made with Type A as the anti-bacterial agent [82]. Moxifloxacin is a type of fluoroquinolone antibiotic and a hydrophobic agent which could effectively act against anaerobic and gram-positive microorganism activity. Moxifloxacin loaded GP has the size of 175 nm with a positive charge of 24 mV. The efficacy of MGP delivered into the anterior segment of the eye via topical administration was evaluated in New Zealand albino rabbits. Similar to the commercial anti-bacterial agent, MoxiGram^®^, MGP showed no irritation, was biocompatible, and was safe on the rabbit cornea and conjunctiva. The anti-bacterial ability was further tested in cultured *Staphylococcus aureus*, and MGP showed a greater effect on inhibition of microbial growth (diameter of the zone of inhibition: 13.36 mm at 12 h and 15.46 mm at 24 h) than the commercial product (MoxiGram^®^) (10.49 mm at 12 h vs. 12.52 mm at 24 h) [82].

Contreras-Ruiz et al. 2013, developed a nanocarrier to treat experimental dry eye (DED) [83]. The DED is usually accompanied by ocular inflammation, which leads to a reduction in mucin production, influences the stability of the tear film, and reduces its capacity to act as a lubricant on the corneal and conjunctival epithelial surfaces during blinking. Mucin 5AC (MUC5AC) is a glycosylated mucin secreted by specialized epithelial cells of the conjunctiva to help improve mucin production in DED. A DNA plasmid carrying MUC5AC gene (pMUC5AC) was loaded into cationized gelatin and chondroitin sulphate to form pMUC5AC loaded GPs (pMUC5AC-GPs, 128 nm positively charged at 37 mV). The gene expression and therapeutic effect of pMUC5AC-GPs for targeting ocular inflammation via topical instillation was investigated in a mouse model of DED. No irritation or edema in the mouse eye was found after topical administration of pMUC5AC-GPs and an improved function of tear production was observed after the treatment [83]. Moreover, inflammatory cytokines, such as interferon γ–induced protein-10 (IP-10) and tumor necrosis factor α (TNFα) were decreased and associated clinical signs such as fluorescein staining and tear production improved. These results show that pMUC5AC-GPs has a great potential as nanomedicine in the treatment of DED.

Recently, Chang et al. 2017, developed self-assembling NPs of type A gelatin and epigalloccatechin-3-gallate (EGCG), a natural anti-angiogenesis component for treating corneal neovascularization (CNV) [14]. To achieve specific targeting of blood vessels, the GPs were surface modified with an arginine-glycine-aspartic acid (RGD) peptide-HA conjugated complex, named GEH-RGD. The RGD peptides were used to direct GPs to target αvβ3 integrin expressed vascular endothelial cells in CNV lesions. The size of GEH-RGD NPs was approximately 168.87 nm and zeta potential was + 19.7 mV. The RGD-HA modified GPs were first tested on human umbilical vein endothelial cells (HUVECs) in vitro. The GEH-RGD NPs were found to enhance cellular uptake and inhibit HUVECs migration and tube formation. In the mouse CNV model, daily topical administration of GEH-RGD NPs (twice a day) revealed fewer vessel formations in the cornea compared to the EGCG solution and non-targeting (GEH) groups. These results suggest that GEH-RGD NPs have great potential for being an active nanomedicine for treating CNV via topical administration [14].

In this section, four types of common polymeric carriers are introduced, and each has its own advantages and drawbacks. For the anterior segment of the eye, the critical factors of drug delivery to consider, regardless of the type of polymer used, should be nanosize, positive surface charge, and mucoadhesive properties to achieve long-term retention on the ocular surface. While for the posterior segment of the eye, more needs to be considered such as penetration ability and controlled release (see as Table 1). The literature on liposome, chitosan, PLGA, and gelatin-based NPs for ocular drug release is summarized in Table 2.

## 5. Possible Routes for Topical Delivery of Polymeric Nanoparticles in the Eye

**Figure 4:**
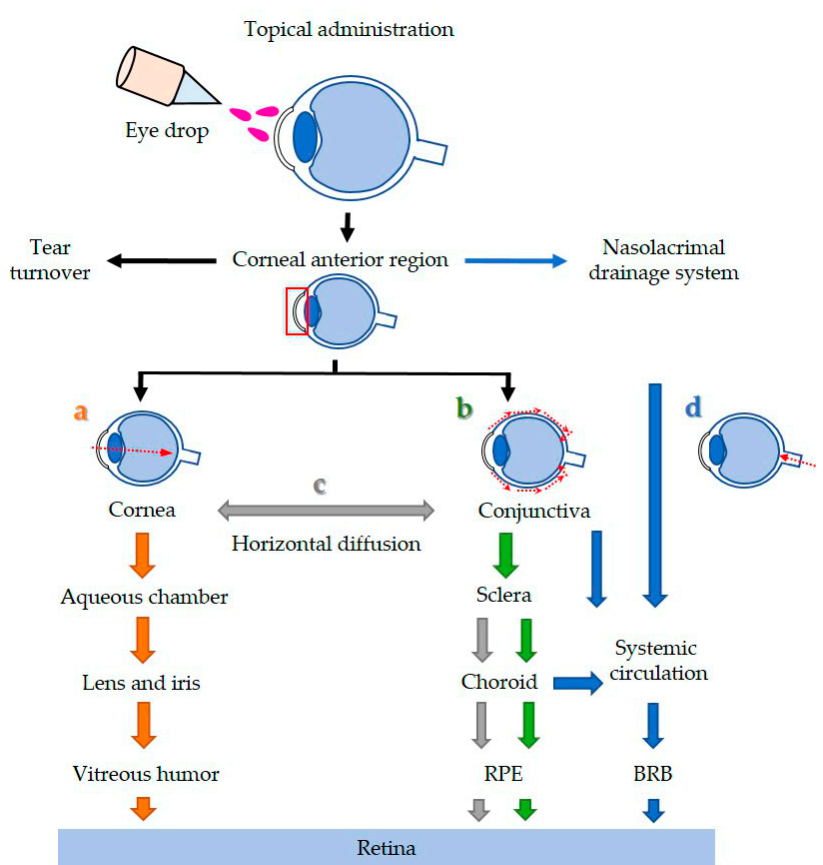
Possible routes of drug delivery to the retina via topical administration. After topical instillation, NPs generally distribute through three main pathways: tear turnover, anterior (cornea/conjunctiva), and the nasolarimal drainage system. RPE: retinal pigment epithelium; BRB: blood–retinal barrier.

In addition to the physical properties of NPs, achieving better therapeutic efficiency in NPs-based ocular drug delivery also depends on how it is delivered. Among all the delivery routes for ocular drug administration (See as Figure 2), topical delivery is still the preferred way because it is non-invasive and has high patient compliance. We have summarized the possible routes of topical delivery to the retina as shown in Figure 4. After topical instillation, NPs are generally distributed through three main pathways: tear turnover, cornea/anterior region, and nasolacrimal drainage. In order to get to the posterior segment of the eye, the dugs will: (a) penetrate through the cornea to the aqueous chamber, then go through the lens/iris to reach the vitreous, and eventually reach the retina. This is a difficult pathway to deliver the agent to the retina. (b) Diffuse through the conjunctiva, sclera, choroid, and finally arrive at the retina. (c) Diffuse horizontally from the cornea to the conjunctiva. (d) Go through the nasolacrimal drainage, the conjunctival blood vessels or choroidal circulation to prevent drugs going from the eye into the systemic circulation, and then returning to the RPE layer by overcoming the BRB [26,49,51,56]. As mentioned earlier, nanoformulation can protect drug activity during transport and achieve drug release in a controlled manner in the desirable ocular tissues [33,35]. However, as yet effective eye drops formulations to deliver drugs to the posterior segment have not been achieved. Therefore, more research is needed to explore different materials and make better nanoformulations for ocular drug delivery.

## 6. Conclusions and Future Aspects

Blindness and visual loss impose substantial lost wellbeing and economic costs. The current treatment modalities for most ocular diseases require frequent intraocular injections for life, with the concomitant risks associated with any invasive intraocular procedure. A non-invasive drug delivery system could potentially eliminate the risks of injection into the eyes. Research has been intensely focused towards development of a new delivery method that can be practiced as a more effective, less-invasive, and long-lasting therapeutic alternative to conventional therapies for ocular damage. Based on these literature reviews, polymeric NPs exhibit a great potential as drug delivery vehicles due to their nanoscale size, biocompatible constituents, and high loading potential for hydrophobic, hydrophilic, and amphiphilic agents. They can potentially overcome the challenges and obstructions of traditional ophthalmic drug applications. These advantages include (1) reducing the frequency of drug administration; (2) overcoming the ocular barrier; (3) protecting the drug activity during transport; and (4) achieving drug release in a controlled manner. Moreover, the targetable NPs can be specifically used to reach the desired tissue or cells, and also minimize the side effects of the drug. Overall, the degradable polymeric nanocarriers formulations come with the promise of some exciting directions in ophthalmology. More clinical studies of degradable polymeric nanocarriers are necessary to provide further information and insights into this great progress in ocular drug delivery.

## Figures and Tables

**Table 1:** Summary of physical properties of nanoparticles influencing its delivery region in the eye.

Property	Effect	Ref.
**Size**	**Anterior** - Particle size <200 nm can be easily taken up in the cornea and conjunctiva	[38,44]
	**Posterior** - Smaller particles (<350 nm) could reach the retina via intravitreal injection. - Hydrophilic NPs (20~80 nm) can pass through the sclera pores, since the scleral water channels/pores are 30~350 nm. - NPs <250 nm are usually easily taken up by retinal cells via endocytosis.	[29,42] [43,45] [47,50] [51]
**Charge**	**Anterior** - Cationic NPs can be attracted to the cornea and conjunctiva due to electrical attraction (Topical delivery)	[30,36]
	**Posterior** - Positively charged NPs tend to get clumped in the vitreous, without diffusing; anionic NPs are able to diffuse to the retina (injection).	[28,33] [38,42] [46,47]

**Table 2:** Summary of common colloidal biodegradable nanoparticles in ophthalmology.

Carriers	Administration Methods	Diseases	Argument	Ref.
Liposomes	Topical administration Subconjunctival injection Intravitreal injection	Dry eye syndrome Fungal keratitis Age-related macular degeneration Glaucoma Autoimmune uveoretinitis	Phospholipid bilayer structure with high biocompatibility, could carry both the hydrophilic or lipophilic drugs, high transfection efficiency, popular and well-researched vehicle.	[53,54,55,56,57,58,59]
Chitosan nanoparticles	Topical administration Corneal stroma injection Subretinal injection	Bacterial endophthalmitis Inherited corneal diseases RPE-associated genetic diseases	Low production costs, the mucoadhesive property could prolonged the drug retention time on the ocular surface, have the ability of breaking through tight junction gaps to overcome the ocular barriers.	[61,62,63,64,65,66,67]
PLGA nanoparticles	Topical administration Intravitreal injection	Corneal inflammatory disorders Uveitis Retinal inflammatory disorders	Well-researched material, superior hydrophilicity, biodegradable and good biocompatibility, could protect the drug from degrading quickly, controlled o drug release.	[68,71,72,73,74,75,76,77]
Gelatin nanoparticles	Topical administration Intravitreal injection	Anterior ocular bacterial disease Dry eye syndrome Corneal neovascularization	Low production costs, component of corneal stroma, polyampholyte, good biocompatibility and biodegradable, easy surface modification, easy and efficient encapsulation of drug molecules or genes.	[14,36,38,78,79,80,81,82,83]
